# Interactions Between Phosphorus, Zinc, and Iron Homeostasis in Nonmycorrhizal and Mycorrhizal Plants

**DOI:** 10.3389/fpls.2019.01172

**Published:** 2019-09-26

**Authors:** Xianan Xie, Wentao Hu, Xiaoning Fan, Hui Chen, Ming Tang

**Affiliations:** ^1^State Key Laboratory of Conservation and Utilization of Subtropical Agro-Bioresources (South China Agricultural University), Guangdong Key Laboratory for Innovative Development and Utilization of Forest Plant Germplasm, College of Forestry and Landscape Architecture, South China Agricultural University, Guangzhou, China; ^2^Department of Plant Pathology, Guangdong Province Key Laboratory of Microbial Signals and Disease Control, College of Agriculture, South China Agricultural University, Guangzhou, China

**Keywords:** phosphorus, zinc, iron, Pi–Zn–Fe interactions, arbuscular mycorrhizal plants

## Abstract

Phosphorus (P), zinc (Zn), and iron (Fe) are three essential elements for plant survival, and severe deficiencies in these nutrients lead to growth retardation and crop yield reduction. This review synthesizes recent progress on how plants coordinate the acquisition and signaling of Pi, Zn, and Fe from surrounding environments and which genes are involved in these Pi–Zn–Fe interactions with the aim of better understanding of the cross-talk between these macronutrient and micronutrient homeostasis in plants. In addition, identification of genes important for interactions between Pi, Zn, and/or Fe transport and signaling is a useful target for breeders for improvement in plant nutrient acquisition. Furthermore, to understand these processes in arbuscular mycorrhizal plants, the preliminary examination of interactions between Pi, Zn, and Fe homeostasis in some relevant crop species has been performed at the physiological level and is summarized in this article. In conclusion, the development of integrative study of cross-talks between Pi, Zn, and Fe signaling pathway in mycorrhizal plants will be essential for sustainable agriculture all around the world.

## Introduction

Inorganic phosphate (Pi), zinc (Zn), and iron (Fe) are three essential macronutrient and micronutrients for the survival and development of all living organisms including mycorrhizal plants and edible crops (Westheimer, 1987; Briat et al., 1995; Marschner, 1995; Salgueiro et al., 2000). These three mineral elements are relatively inaccessible to plants and crops because of their low solubility and relative immobilization in the agricultural soils (Lopez et al., 2000; Hirsch et al., 2006). Crops are therefore subjected to Pi, Zn, and Fe deficiencies, which can adversely impact multiple metabolic processes in cells. Nevertheless, plants have evolved a number of strategies to cope with low Pi, Zn, and Fe availabilities, including development of a mycorrhizal symbiosis (Karandashov and Bucher, 2005; Smith and Read, 2008), conversion of metabolism, remodeling of root morphology, secretion of root exudates, and induction of the high-affinity transport systems.

In recent decades, the effects of Pi, Zn, and Fe deficiencies on crop yield and quality have become a global concern due to the issues of food availability and malnutrition (Abelson, 1999; Neset and Cordell, 2012; Shahzad et al., 2014). To guarantee the sustainable food source for the growing population, worldwide agriculture has become dependent on the massive use of Pi, Zn, and Fe fertilizers for improving crop yield and quality. Nevertheless, this strategy has adverse long-term economic and ecological impacts. Development of sustainable agricultural practices will require crops with improved Pi, Zn, and Fe nutrition in order to reduce the application of these fertilizers. The novel plant genotypes with high-efficiency nutrient use are genetically desired in an appropriate way to fit the lower input into the environment. Despite the importance of these issues, the biological interactions between P, Zn, and Fe elements still remain incompletely studied, and our understanding is limited of how various signaling pathways are induced in response to nutrient availability and how these changes are integrated with relation to other nutrients (Briat et al., 2015). On the other hand, some key genes involved in the acquisition and distribution of macronutrient and micronutrients in nonmycorrhizal and mycorrhizal plants have been identified (Javot et al., 2007a; Gojon et al., 2009; Pilon et al., 2009; Giovannetti et al., 2014; Watts-Williams and Cavagnaro, 2018), and their expression in response to nutrient status has started to be elucidated (Schachtman and Shin, 2007; Giehl et al., 2009; Liu et al., 2009; Hindt and Guerinot, 2012).

Approximately 72% of terrestrial vascular plant species are capable of establishing symbiotic mutualistic associations with obligate biotrophic soil-borne arbuscular mycorrhizal fungi (AMF) from the phylum Glomeromycota (Remy et al., 1994; Schüßler et al., 2001; Bonfante, 2018). The endosymbiotic associations between plants and AMF, namely, arbuscular mycorrhizas (AM), are widespread in terrestrial ecosystems (Parniske, 2008). In AM symbiosis, the fungal symbiont provides mineral nutrients to the plant and in return obtains sugars and lipids (Smith and Read, 2008; Jiang et al., 2017), and thus, this symbiosis has significant contribution to plant productivity and ecosystem function (van der Heijden et al., 1998).

AM symbiosis not only is capable of significantly improving the acquisition of macronutrients such as Pi, N, and S to host plant (Ames et al., 1983; Smith et al., 2003; Smith and Read, 2008; Allen and Shachar, 2009; Leigh et al., 2009; Sieh et al., 2013) but also facilitates the uptake and translocation of micronutrients such as Zn and Fe in the soil–AMF–plant continuum (Caris et al., 1998; Liu et al., 2000; Chen et al., 2003; Farzaneh et al., 2011; González et al., 2016). The acquisition of Pi, N, and S in AM symbiosis through a specific symbiotic uptake pathway has been extensively described (Rausch et al., 2001; Harrison et al., 2002; Javot et al., 2007b; Chen et al., 2007; Guether et al., 2009; Yang et al., 2012; Giovannetti et al., 2014; Volpe et al., 2016). However, very few studies have been undertaken to uncover the molecular mechanisms underlying the uptake and homeostasis of Zn and Fe from AM fungus *Rhizophagus irregularis* to the plant (González et al., 2005; Tamayo et al., 2014; Tamayo et al., 2018), and the impact of this symbiosis on Zn and Fe homeostasis in plant is far from being understood (Chorianopoulou et al., 2015; Watts-Williams et al., 2015). A very recent study has revealed the involvement of AM-modified *ZmNAS1*, *ZmNAS3*, and *ZmYS1* genes in the regulation of Fe homeostasis in mycorrhizal maize through sulfate deficiency signaling (Chorianopoulou et al., 2015), suggesting the existence of a cross-talk between S and Fe homeostasis in mycorrhizal symbiosis. Nevertheless, the molecular basis of the double or tripartite interactions between Pi, Zn, and Fe homeostasis in AM symbiosis is still lacking in mycorrhizal plants. Therefore, it is of biological significance to decipher the mechanisms of coordinating the Pi, Zn, and Fe deficiency signaling in AM symbiosis and consequently profit mycorrhizal plant growth and fitness during multiple Pi–Zn–Fe deficiency stresses.

In such context, the aim of this review is to summarize current knowledge on cross-talk between Pi and Zn, Pi and Fe, Zn and Fe, and tripartite Pi–Zn–Fe homeostasis in both nonmycorrhizal and mycorrhizal plants. Additionally, Pi (or Fe) nutrition is also affected by the interaction between Zn and Fe (or Pi) in plants, such as *Arabidopsis* and rice. The MYB transcription factor (TF) PHR1 acting as a potential integrator of Pi, Zn, and Fe nutrient signals to regulate mineral nutrition in plants is discussed. Moreover, a novel role of the OsPHO1;1 in Fe transport through integrating Pi and Zn deficiency signaling is proposed, and these complicated nutritional interactions are presented, with a focus on the emerging roles of nutrient transporters in mycorrhizal plants.

### Membrane Transporters and Their Roles in Mineral Uptake and Homeostasis in Plants

In plants, Pi, Zn, and Fe are acquired at the root periphery in the form of free ions (Guerinot, 2000; Curie et al., 2001; Vert et al., 2002; Nussaume et al., 2011; Milner et al., 2013), and the uptake and translocation of these minerals in plants involve multiple and complex transport systems.

The Pi is taken up at the root system *via* the high-affinity Pi:H^+^ symporters belonging to members of the PHT1 family (Schachtman et al., 1998; Bucher, 2007; Nussaume et al., 2011). The *Arabidopsis*, rice, soybean, and tomato genomes harbor 9, 13, 14, and 8 members of the PHT1 family, respectively (Paszkowski et al., 2002; Poirier and Bucher, 2002; Fan et al., 2013; Chen et al., 2014). Some of these *Pht1* genes are predominantly expressed in roots, and the encoded proteins function as high-affinity Pi uptake transporters (Muchhal et al., 1996; Shin et al., 2004; Remy et al., 2012; Sun et al., 2012). Nevertheless, the transcripts of *Pht1* genes are also detected in shoots (including vegetative and reproductive tissues), implicating their role beyond Pi uptake at the root surface (Mudge et al., 2002; Nagarajan et al., 2011; Chen et al., 2014). In *Arabidopsis*, five out of nine *Pht1* members have been functionally characterized by genetic approaches. Earlier work reported that both AtPT1 and AtPT4 transporters contributed to Pi uptake in *Arabidopsis thaliana* under both low and high Pi levels (Shin et al., 2004). However, the double mutant *pht1;1Δpht1;4Δ* showed a more pronounced reduction in Pi acquisition relative to wild-type from both low and high Pi environments, suggesting redundant functions of these two Pi transporters (Shin et al., 2004). Nevertheless, Nagarajan et al. (2011) showed that the AtPT5 could mobilize Pi between the source and sink organs for Pi homeostasis in *A. thaliana*. Recently, it was demonstrated that AtPT8 and AtPT9 transporters act in a redundant manner during Pi uptake in *Arabidopsis* seedlings during Pi starvation (Remy et al., 2012). These results indicated the compensatory effects of root Pi uptake and shoot Pi accumulation between the four *Arabidopsis* Pi transporters AtPT1, AtPT4, AtPT8, and AtPT9 during Pi deficiency. In rice (*Oryza sativa*), a total of 13 members of the PHT1 family have been identified (Goff et al., 2002), and 10 out of 13 genes had been well characterized in *O. sativa* by reverse genetics. The constitutively expressed OsPT1 mediates Pi translocation in shoots and also induces root hair growth in rice during Pi-repletion (Sun et al., 2012). Ai et al. (2009) demonstrated that *OsPT2* was transcriptionally induced in roots under Pi deficiency and functioned in Pi translocation in rice, while OsPT3 mediated Pi uptake, translocation, and remobilization in rice under extremely low Pi regimes (Chang et al., 2019). OsPT4 not only facilitated Pi mobilization but also played a pivotal role in embryo development (Zhang F et al., 2015), whereas OsPT6 displayed a broad role in Pi acquisition and translocation throughout the plant (Ai et al., 2009). It was observed that the high-affinity Pi transporter gene *OsPT8* was involved in Pi homeostasis in rice (Jia et al., 2011). However, Wang et al. (2014b) found that OsPT9 and OsPT10 redundantly functioned in Pi uptake under both low and high Pi conditions. OsPT11 and OsPT13 were exclusively induced in arbusculated cells and non-redundantly regulated the arbuscular mycorrhizal symbiosis in rice (Paszkowski et al., 2002; Yang et al., 2012). The current understanding of the Pi transport activities of Pht1 transporters and their complex regulation in plants has been well documented and intensively summarized in multiple reviews in recent years (Poirier and Bucher, 2002; Bayle et al., 2011; Lin et al., 2013; Chen et al., 2015; Poirier and Jung, 2015; Gu et al., 2016).

For Zn^2+^ acquisition in roots, transmembrane transporters belonging to the ZIP (ZRT and IRT-like protein) family are considered to be the primary Zn^2+^ uptake transporters, which have been identified in both dicotyledons and monocotyledons (Eng et al., 1998; Maser et al., 2001; López et al., 2004; Palmer and Guerinot, 2009; Lee et al., 2010; Tiong et al., 2014). Some ZIP family transporters preferentially localize to the plasma membrane of root epidermal cells and deletion, or overexpression of these genes results in plants that accumulate less or more Zn^2+^ than do wild-type plants, respectively. This is indicative of their roles in Zn^2+^ acquisition at root–soil interface (Lee et al., 2010; Milner et al., 2012). In *Arabidopsis*, the plasma membrane-localized AtIRT1, belonging to the ZIP gene family, is involved in Zn^2+^ uptake at root epidermal cells (Henriques et al., 2002; Vert et al., 2002; Barberon et al., 2011). The well-characterized ZIP gene *IRT3* is transcriptionally induced in response to Zn^2+^ deficiency and confers increased shoot Zn^2+^ accumulation when overexpressed in *Arabidopsis* (van de Mortel et al., 2006; Sinclair and Krämer, 2012). Moreover, AtIRT3 is localized to the plasma membrane where it transports Zn^2+^ across the plasma membrane into the cell (Lin et al., 2009). In rice, the node-localized transporter, OsZIP3, is responsible for unloading Zn^2+^ from the xylem as well as Zn^2+^ distribution to the developing tissues (Sasaki et al., 2015), whereas the OsZIP4 located in the phloem cells acts as a Zn^2+^ transporter that may be responsible for Zn^2+^ translocation within plant (Ishimaru et al., 2005). Other *Arabidopsis* and rice ZIP family members involved in Zn^2+^ uptake and homeostasis are barely known, and therefore, further works need to determine their precise roles in plants.

**Figure 1:**
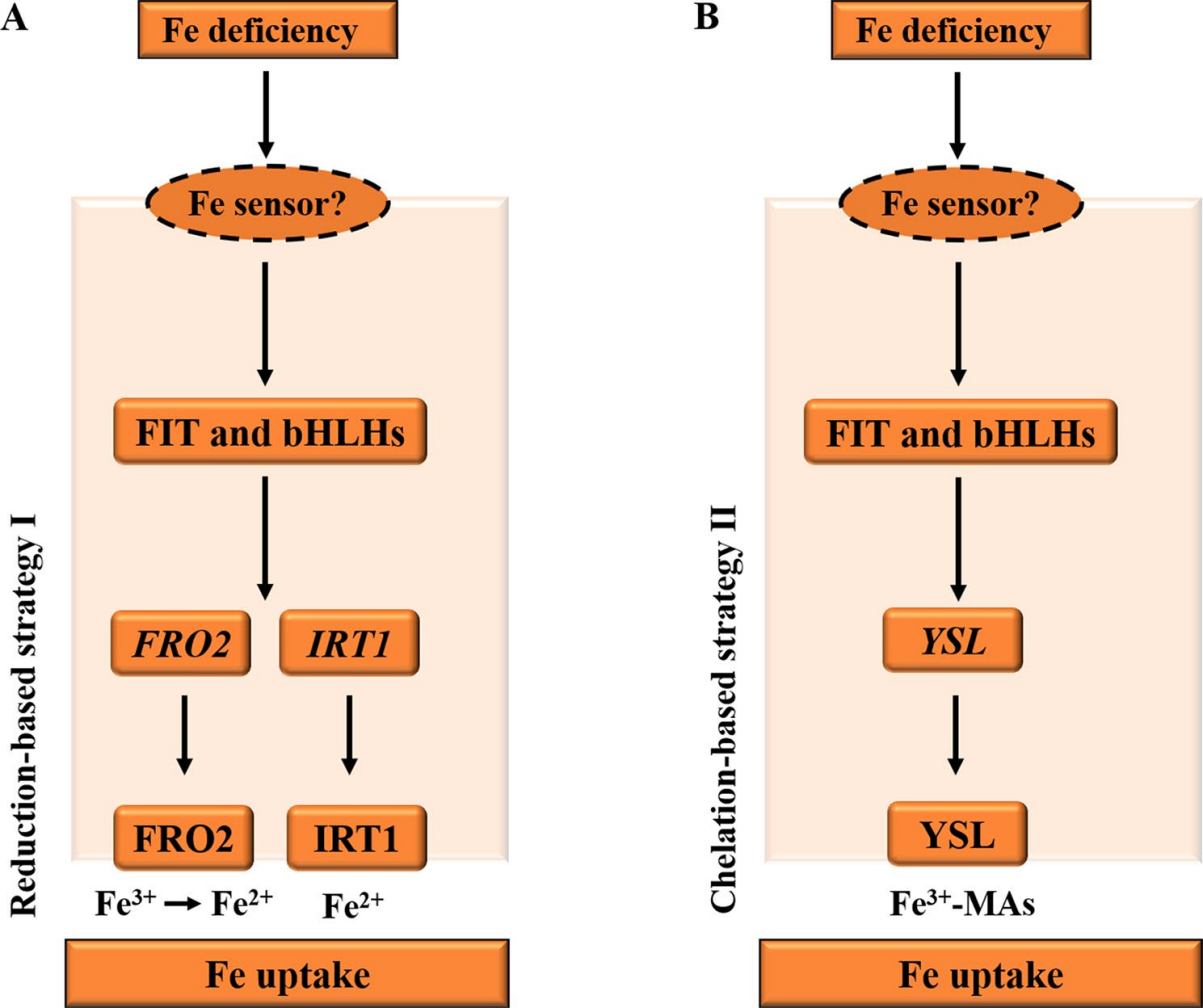
Diagrams illustrating the iron deficiency response in *Arabidopsis* and *Graminacea* plants. **(A)** Under Fe deficiency, in *Arabidopsis* roots, the FIT and bHLH transcription factors, such as bHLH38 and bHLH39 in *Arabidopsis*, are activated by an unknown PM iron sensor in order to bind to the promoters of the iron-responsive genes. Subsequently, the induction of FRO2 and IRT1 activity is co-regulated in response to iron deficiency through the reduction-based strategy I for iron uptake. Iron is firstly reduced from ferric (Fe^3+^) to ferrous (Fe^2+^) iron by the reductase FRO2. Fe^2+^ is then imported into the root cell by the metal transporter IRT1. **(B)** Under Fe deficiency, the FIT and bHLH transcription factors are activated to induce the strategy II Fe uptake system in *Graminacea* plant roots. Fe^3+^–MAs are transported into the root cell by the YSL. PM, plasma membrane; YSL, yellow stripe like; IRT1, iron-regulated transporter 1. The arrows refer to the positive interactions, while the question marks indicate the unknown iron sensor.

Iron (Fe) from the soils enters the root cells through two distinct strategies (**Figure 1**), according to non-Graminacea plants (strategy I) and Graminacea plants (strategy II). In strategy I plants, the ferric iron (Fe^3+^) is reduced in the ferrous iron (Fe^2+^) prior to uptake into the root epidermal cells (Morrissey and Guerinot, 2009; Conte and Walker, 2011). For example, in *Arabidopsis*, under Fe deficiency, the FIT and bHLH TFs, bHLH38 and bHLH39, are activated in roots and bind to the promoters of the iron-responsive genes. Subsequently, the induction of ferric reductase oxidase 2 (FRO2) and IRT1 activity is co-regulated in response to iron deficiency through the reduction-based strategy I for iron uptake (**Figure 1A**), while iron in rhizosphere is firstly solubilized by the activated H^+^-ATPase AHA2 and is then reduced from ferric (Fe^3+^) to ferrous (Fe^2+^) iron by the reductase FRO2 (Ivanov et al., 2012). Fe^2+^ is then imported into the root cell by the metal transporter IRT1 (Vert et al., 2002).

In strategy II plants, the ferric iron (Fe^3+^) is first chelated by interaction with mugineic acids (MAs) (**Figure 1B**), and then these Fe^3+^–MA complexes are taken up into root cells by plasma membrane-localized transporter proteins (Curie et al., 2001). For example, in maize, under iron deficiency, MAs are synthesized in root cells by nicotianamine synthase (NAS), NA aminotransferase (NAAT), and deoxymugineic acid synthase (DMAS); and MAs are secreted into rhizosphere by transporter of MA family phytosiderophores1 (TOM1) (Li et al., 2018). Then, Fe^3+^–MAs are transported into root cells by the transmembrane transporter yellow stripe 1-like (YSL) (Curie et al., 2001).

After their uptake at the root surface, these minerals can be transported to the vacuoles. Alternatively, Pi, Zn^2+^, and iron can undergo symplastic journey towards the root xylem for movement upward to the aerial tissues. For Pi, phosphate exporters PHO1 and PHO1;H1 have been identified as important components in the long-distance transfer of Pi from roots to shoots (Poirier et al., 1991; Hamburger et al., 2002; Stefanovic et al., 2011; Kisko et al., 2015).

For Zn^2+^, two plasma membrane transporters AtHMA2 and AtHMA4 belonging to P_1B_-ATPase subfamily played key roles in Zn loading into the xylem and root-to-shoot translocation of Zn^2+^ in *Arabidopsis* (Hussain et al., 2004; Verret et al., 2004; Hanikenne et al., 2008; Wong et al., 2009). NA had been proposed to form stable complexes with Zn and to play an important role in Zn^2+^ movement in the xylem and phloem (Stephan and Scholz, 1993). NAS genes were induced under Zn^2+^ deficiency (Wintz et al., 2003) and were functionally involved in the intercellular movement and long-distance transport of Zn^2+^ in *A. thaliana* (Takahashi et al., 2003). Overexpression of *AhNAS2* gene in roots contributed to Zn^2+^ hyperaccumulation of *Arabidopsis halleri* (Deinlein et al., 2012). Interestingly, the constitutive expression of NAS genes from other plant species caused an increase in Zn^2+^ translocation and accumulation in polished rice grains (Masuda et al., 2009; Lee et al., 2011), illustrating the significant importance of the NAS proteins in the Zn^2+^ translocation in plants. The major facilitator superfamily (MFS) transporter (Pao et al., 1998), zinc-induced facilitator 1 (ZIF1), was shown to contribute to Zn^2+^ tolerance in *Arabidopsis* (Haydon and Cobbett, 2007), and tonoplast-localized ZIF1 proteins have been implicated in vacuolar Zn^2+^ sequestration (Arrivault et al., 2006; Kawachi et al., 2009). Under Zn^2+^ deficiency, the vacuole-stored Zn^2+^ was remobilized (Lanquar et al., 2004) to the cytosol. Natural resistance-associated macrophage protein (NRAMP) family members played roles in heavy metal transport in plants (Belouchi et al., 1995; Thomine et al., 2000). *Arabidopsis* NRAMP4 localized to the vacuolar membrane and associated with Zn^2+^ remobilization (Lanquar et al., 2004). Whether other members of the NRAMP family could contribute to Zn^2+^ remobilization in plants remains unknown.

For Fe, many transporters and soluble proteins responsible for Fe long-distance transfer and distribution have been characterized in recent years (Morrissey and Guerinot, 2009; Kobayashi and Nishizawa, 2012). In *Arabidopsis*, the AtFRD3 protein, which is a member of the multidrug and toxic compound extrusion (MATE) family, functions during efflux of citrate into xylem and is responsible for Fe long-distance transport from root xylem to shoots (Green and Rogers, 2004; Durrett et al., 2007; Magalhaes et al., 2007), whereas the rice OsFRDL1, the ortholog of FRD3, maintains the Fe^3+^ levels in the xylem sap (Yokosho et al., 2009). YSL transporters play a significant role in the transportation and distribution of Fe through the phloem (Curie et al., 2009) and are also involved in loading Fe from old leaves to flowers and developing seeds (Kobayashi and Nishizawa, 2012). Moreover, in rice, OsYSL2 and OsYSL15 may coordinate the long-distance Fe transport from root to shoot to seed (Koike et al., 2004; Inoue et al., 2009). In addition to YSLs, the iron transport protein ITP, which is an Fe-binding dehydrin in the phloem sap, helps promote Fe^3+^ mobility within the phloem of *Ricinus communis* (Kruger et al., 2002). Plant NAS genes are also required for long-distance Fe transport. For instance, in *Arabidopsis*, AtNAS2 and AtNAS4 may be involved in Fe translocation from roots to shoots (Klatte et al., 2009). Interestingly, the rice *OsIRT1* are highly expressed in the companion cells of phloem under Fe deficiency (Ishimaru et al., 2006), and it is possible that the corresponding encoding protein OsIRT1 could transport Fe^2+^ into the phloem prior to being chelated by NA. More recently, the involvement of OsPHO1;1 in the Fe loading into the root xylem has been reported, where it may affect overaccumulation of Fe in roots of the *Ospho1;1* mutant under Pi and Zn deficiency (Saenchai et al., 2016).

### Nutrition Sensing and Signaling in Plants

**Figure 2:**
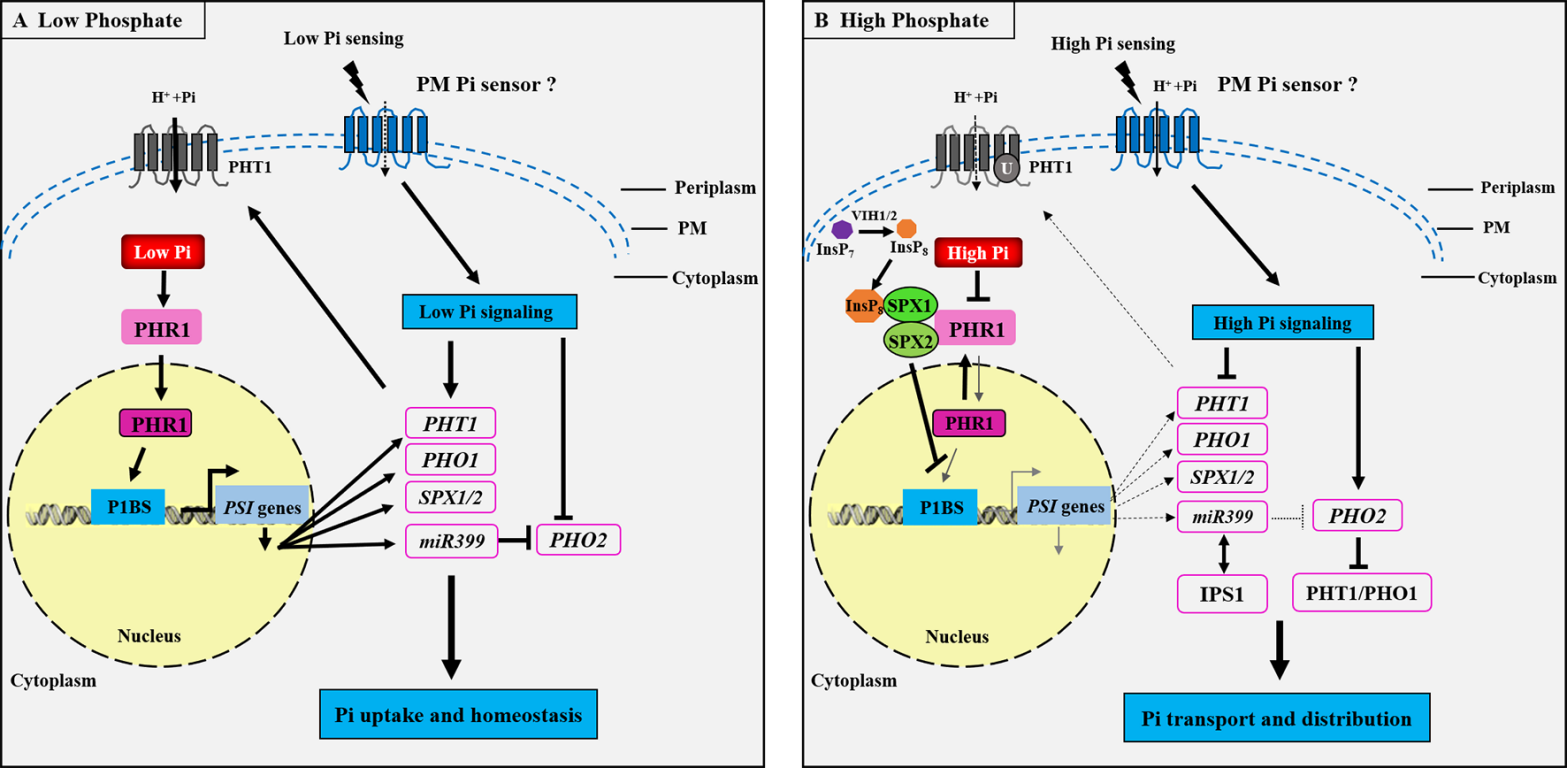
Schematic representation of the phosphate (Pi) signaling pathway essential for plant adaptation to low Pi concentration. Under Pi deficiency **(A)**, a set of phosphate starvation-induced (*PSI*) genes are transcriptionally activated through binding of the transcription factor PHOSPHATE STARVATION RESPONSE 1 (PHR1) to the cis-element (P1BS) present in the promoter region of the *PSI* genes, and subsequently *PHT1* and *PHO1* mRNAs are induced to be necessary for Pi uptake and translocation in roots. The *SPX1/2* and *miR399* genes are also activated by PHR1 during Pi starvation. miR399 inhibits the ubiquitin E2 conjugase PHO2 in order to maintain the PHT1 protein activity at the PM. It could be proposed that the Pi signaling is activated for sensing external low Pi through an unknown PM Pi sensor, which induces the low Pi responsive genes *PHT1*, *PHO1*, *SPX1/2*, and *miR399*, whereas the *PHO2* is repressed, thus activating the Pi regulatory pathway to modulate Pi uptake and homeostasis. Under high Pi concentration **(B)**, the Pi signaling pathway is repressed, the diphosphoinositol pentakisphosphate kinases VIH1 and VIH2 function redundantly to synthesize InsP_8_, and InsP_8_ can directly bind to the SPX domain of SPX1 and is essential for the interaction between SPX1/2 and PHR1. This interaction leads to the inhibition of PHR1 binding to the *cis*-element P1BS present in the promoter region of the *PSI* genes. Thus, the *PSI* genes, including *PHT1*, *PHO1*, *SPX1/2*, and *miR399*, are transcriptionally repressed, while the *PHO2* is activated to be responsible of the ubiquitination of PHT1 and PHO1 proteins to promote Pi transporters degradation. *IPS1* encodes a non-coding RNA and enables post-transcriptional regulation under high Pi through RNA mimicry. IPS1-miR399 matching thus results in the inhibition of the miR399 activity to target PHO2. It is also predicted that there may exist an unknown PM Pi sensor responsible for high Pi sensing. PM, plasma membrane. The arrows and flat-ended lines refer to the positive and negative interactions, respectively. The dotted arrows represent the repression processes.

Considerable advances have been made in studying the molecular mechanisms underlying Pi, Zn, and Fe sensing and signaling in plants in recent decades (Abel et al., 2002; Chiou and Lin, 2011; Assuncão et al., 2013; Kobayashi and Nishizawa, 2014; Zhang et al., 2014). Nevertheless, the cross-talks between these signaling pathways integrating the tripartite interaction among Pi, Zn, and Fe homeostasis remains poorly understood (Briat et al., 2015; Saenchai et al., 2016). How Pi homeostasis is regulated in plants has already been documented in numerous studies, and plant Pi sensing seems to be conserved in flowering plants, with several signaling networks having been proposed (Rouached et al., 2010; Wang et al., 2014a). The defined mechanism is the systemic Pi signaling cascade, which contains the MYB TF PHR1, the miRNA399, and the ubiquitin E2 conjugase PHO2 components (Bari et al., 2006; Pant et al., 2008). Generally, the well-characterized PHR1–PHO2–miRNA399 signaling pathway controls the expression of most Pi starvation-induced (*PSI*) genes in plants (**Figure 2**). In response to low Pi, *miRNA399* is transcriptionally induced by PHR1 activity (**Figure 2A**) and then mediates shoot-to-root Pi signaling *via* the phloem, where it targets the mRNA of *PHO2* (Lin et al., 2008; Pant et al., 2008). The inhibition of *PHO2* leads to an increase in the PHR1-dependent expression of root Pi transporters that include the members of PHT1 and PHO1, and hence an increase in Pi acquisition in roots and Pi translocation to shoots (Bari et al., 2006; Lin et al., 2008). Under high Pi conditions, *miRNA399* is down-regulated due to the inhibition of PHR1 activity (**Figure 2B**), and the PHO2–miR399 pathway in roots is dysfunctional through target mimicry between miR399 and PHR1-dependent IPS1 (Franco et al., 2007). Target genes of PHR1 are also reduced at transcriptional level, and PHO2 protein is activated to facilitate the degradation of Pi transporters.

Recently, the SPX domain-containing proteins have been proposed to function as the intracellular Pi sensors for sensing cellular Pi levels and controlling Pi homeostasis in both monocotyledonous and dicotyledonous plants. In *Arabidopsis*, the PHR1-dependent *AtSPX1* gene is transcriptionally induced under Pi deficiency (**Figure 2A**), while the AtSPX1 protein can interact with the AtPHR1 at the protein level under Pi sufficiency, inhibiting AtPHR1 binding to P1BS *cis*-element (GNATATNC) (Puga et al., 2014). Similarly, in rice, OsSPX1 and OsSPX2 inhibit Pi deficiency response through interaction with OsPHR2 in a Pi-dependent manner (Wang et al., 2014a), involvement of SPX proteins in the Pi sensing, and signaling mechanisms in plants (**Figure 2B**). Very recently, it has demonstrated that both the diphosphoinositol pentakisphosphate kinases (PPIP5K) VIH1 and VIH2 function redundantly to synthesize the inositol pyrophosphate (InsP_8_) (see **Figure 2B**), and InsP_8_ can directly bind to the intracellular Pi sensor SPX1 to control Pi homeostasis in *Arabidopsis* during Pi repletion (Dong et al., 2019). This study revealed that InsP_8_ acts as an intracellular phosphate signal in plants. The next major challenge in this field is to unmask the extracellular Pi sensor sensing.

In plants, how Zn-deficient signal is sensed, relayed, and integrated into a signal response remains elusive. Nevertheless, a first working model of Zn deficiency signaling has been proposed by Assuncão et al. (2013). Two bZIP TFs, bZIP19/23, have been identified in Zn homeostasis *via* the regulation of target genes, including the members of ZIP family (Guerinot, 2000; Assuncão et al., 2010a) for root Zn transport and *NAS2*/*4* for NA synthesis (Assuncão et al., 2010b), since the shoots appear to the first organ to sense the Zn deficiency and then transmit the signal to the roots where these ZIP transporters are activated (Assuncão et al., 2013). This observation has led to the proposal of the existence of unknown long-distance Zn deficiency signaling molecules. Additionally, a ZDRE element (RTGTCGACAY) is present in the promoter regions of both the *ZIP* and *NAS* genes in response to Zn deficiency (Assuncão et al., 2010; Assuncão et al., 2010b).

How the Fe status of plant is sensed and how this signal is transmitted to the transcriptional networks for Fe acquisition and response are currently areas of great interest in the field of Fe homeostasis in plants (Briat et al., 2015). A major goal is to find a master Fe sensor controlling Fe homeostasis in plants (Hindt and Guerinot, 2012). Some degree of progress towards these aims has been achieved by exploiting members of the basic helix-loop-helix (bHLH) TF family (Hindt and Guerinot, 2012; Ivanov et al., 2012; Kobayashi and Nishizawa, 2012; Moran et al., 2014). Also, hemerythrin motif-containing RING and zinc-finger proteins HRZ1/2 and its ortholog E3 ligase BTS that have been recently characterized in both the monocotyledonous and dicotyledonous plants, respectively (Kobayashi and Nishizawa, 2014). The tomato FIT is referred as to FER (Ling et al., 2002; Brumbarova and Bauer, 2005), and FER mutant *fit-1* repressed about 50% of Fe deficiency-induced genes in roots (Colangelo and Guerinot, 2004). A second PYE bHLH protein is exclusively induced in roots under Fe deficiency. The *pye-1* mutant line is sensitive to low Fe. In *pye-1* mutant, three Fe transport-related genes, *NAS4*, *FRO3*, and *ZIF1*, are strongly induced under Fe deficiency and are identified as the targets of PYE (Long et al., 2010). In addition, *Arabidopsis* bHLH104 and ILR3 play crucial roles in the regulation of Fe deficiency responses through targeting other bHLH genes and PYE expression (Zhang et al., 2015). The overexpression lines of rice iron-related TF 2 (OsIRO2), ortholog of *Arabidopsis* bHLH38/39, showed both enhanced Fe uptake and transportation to seeds (Ogo et al., 2007; Ogo et al., 2011). Furthermore, a PYE homologous protein OsIRO3 is induced under Fe deficiency, whereas it is a negative regulator of Fe deficiency responses due to the hypersensitivity to Fe deficiency and the inhibition of genes up-regulated by Fe deficiency (Zheng et al., 2010). Recently, rice bHLH133 was identified to play an important role in the regulation of Fe translocation from roots to shoots (Wang et al., 2013). On the other hand, BTS and its orthologs HRZ1/2 could negatively regulate Fe acquisition, accumulation of Fe, and tolerance to Fe deficiency in rice *HRZ1/2* mutants (Kobayashi et al., 2013).

### Interactions Between P, Zn, and Fe Homeostasis in Plants

Cross-talks between macronutrient and micronutrients in plants have long been recognized, and these interactions are understood to some extent. Hence, we here emphasize the interactions between Pi, Zn, and iron (Fe) homeostasis at the physiological and molecular levels. The interaction between two nutrients homeostasis has been observed in crop species.

The interaction between Pi and Zn homeostasis in plants is relatively well understood. Pi deficiency results in overaccumulation of Zn in shoots, and inversely, Zn deficiency leads to overaccumulation of Pi in the aerial part of plants (Reed, 1946; Cakmak and Marschner, 1986; Huang et al., 2000; Bouain et al., 2014a; Khan et al., 2014; Ova et al., 2015). In addition to the well-known antagonistic effect of Pi and Zn nutrition in plants, there is some evidence of similar physiological interactions between Pi and Fe nutrition (Zheng et al., 2009), and between Zn and Fe nutrition (Haydon et al., 2012) as well. Pi acquisition in both roots and shoots is promoted under Fe deficiency, and conversely, Pi deficiency significantly increases Fe availability within the plants (Misson et al., 2005; Hirsch et al., 2006; Ward et al., 2008; Zheng et al., 2009; Briat et al., 2015). Fe deficiency leads to an accumulation of Zn, while an excess Zn causes physiological Fe deficiency (Haydon et al., 2012; Shanmugam et al., 2012; Briat et al., 2015).

**Figure 3:**
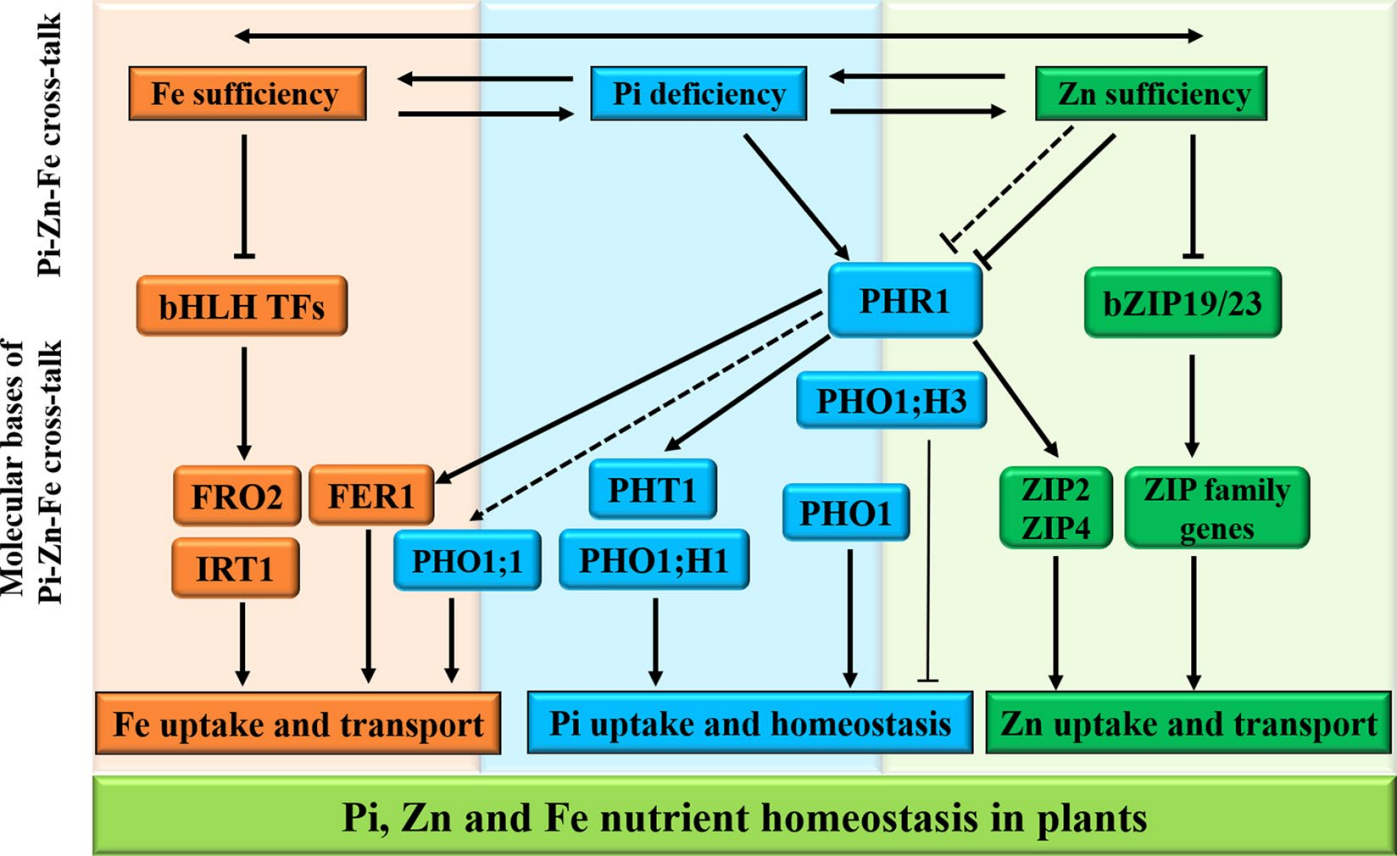
Schematic representation of Pi, Zn, and Fe homeostasis interactions in plants. The cross-talks between phosphate (Pi), zinc (Zn), and iron (Fe) nutrients are shown at the physiological level by two-way arrows. For the molecular bases of the Pi–Zn–Fe cross-talks, the PHR1 acts as a potential integrator of Pi, Zn, and Fe nutrient signals in plants. Firstly, the PHR1 was defined as a key regulator of the expression of Pi transporters PHT1 and PHO1; H1 through the PHR1–miR399–PHO2 pathway (see **Figure 2**). Secondly, *ZIP2* and *ZIP4* genes, belonging to plant ZIP gene family, are transcriptionally induced *via* the activated PHR1 transcription factor binding to the P1BS (GNATATNC) sequences found in the promoter regions of their genes. Under Zn sufficiency, the bZIP19/23 transcription factors are inactivated, and bZIP19/23-mediated Zn regulatory pathways repress the plant ZIP gene family transporters in order to regulate Zn homeostasis. On the other hand, *PHO1;H3*, which is transcriptionally down-regulated by high Zn supply, and the PHR1 and PHO1 proteins contribute to the Pi–Zn nutrient homeostasis cross-talk. In addition, the transcriptional activation of some genes involved in maintaining Fe homeostasis is also shown to be PHR1-dependent manner, including the *FER1* gene encoding the Fe storage protein ferritin, and the *PHO1;1* gene encoding Pi transporter. The arrows and flat-ended lines indicate the positive and negative interactions, respectively.

In plants, the intricate cross-talks between the homeostasis of macronutrients and micronutrients have recently become clear (Briat et al., 2015), and evidence of a complex tripartite interaction between Pi, Fe, and Zn nutrients for maintenance of Pi homeostasis in *Arabidopsis* has been described (Rai et al., 2015). In addition, Saenchai et al. (2016) have also provided evidence that iron transport in rice is regulated by integration of Pi and Zn deficiencies, highlighting the presence of tripartite cross-talk between Pi, Zn, and Fe homeostasis for better plant survival and fitness (**Figure 3**).

### Molecular Evidence for Pi, Zn, and Fe Interactions in Plants

Although the cross-talks between Pi, Zn, and Fe homeostasis have been reported in many plant species (Briat et al., 2015), the molecular basis and biological significance of these nutritional interactions remain thus far largely unknown. It can be first achieved through transcriptomic and genetic analyses of Pi-, Zn-, or Fe-deficient plants (Hammond et al., 2003; Wu et al., 2003; Misson et al., 2005; van de Mortel et al., 2006; Zheng et al., 2009; Bustos et al., 2010; Thibaud et al., 2010; Rouached et al., 2011b; Pineau et al., 2012; Khan et al., 2014; Moran et al., 2014; Rai et al., 2015; Saenchai et al., 2016).

Zn deficiency activates the transcription of numerous Pi-related genes (van de Mortel et al., 2006), while Pi deficiency up-regulates the expression of genes involved in Zn and Fe homeostasis (Misson et al., 2005; Bustos et al., 2010). More recently, several reports have proposed that PHR1, PHO1 and PHO1;H3 are coordinatively involved in the homeostasis between Pi and Zn in *Arabidopsis* (Bouain et al., 2014b; Khan et al., 2014; Kisko et al., 2015), reinforcing the interaction between Pi and Zn signaling at the molecular level (**Figure 3**).

In the absence of Pi, plants induce the expression of genes in response to sufficient Fe, whereas Pi-starvation plants reduce the transcripts of genes in response to Fe deficiency (Misson et al., 2005; Müller et al., 2007; Thibaud et al., 2010). Reciprocally, Fe deficiency alters the transcription of Pi-related genes (Zheng et al., 2009; Moran et al., 2014). Genome-wide analysis further reveals 547 and 579 overlapping genes regulated by both Pi and Fe deficiency in rice and *Arabidopsis* roots, respectively (Zheng et al., 2009; Li and Lan, 2015). In these cases, the expression of *FER1* gene encoding Fe storage protein ferritin is in response to Pi starvation mediating by PHR1 (**Figure 3**) and Fe excess (Petit et al., 2001; Bournier et al., 2013), and *NAS3* and *YSL8* genes responsible for Fe homeostasis are also induced upon Pi starvation in plants (Bustos et al., 2010). However, the *IRT1/2*, *FRO3/6*, and *NAS1* genes are repressed in response to Fe deficiency in Pi-deficient plants. Recently, it has reported that the *Arabidopsis phr1* × *phl1* double mutant influenced Fe distribution and Fe-related gene expression (Bournier et al., 2013; Briat et al., 2015), suggesting that PHR1 and PHL1 may integrate Fe and Pi nutrient signals. The high-affinity copper transport protein COPT2 acts as a key player in the interaction between Pi and Fe deficiency signaling in *Arabidopsis* (Perea et al., 2013). COPT2 may play a dual role under Fe deficiency. It participates in copper uptake and distribution in Fe-limited roots to minimize iron loss. On the other hand, loss of COPT2 function exacerbates Pi starvation responses in *Arabidopsis* plants. These findings open new approaches to mitigate iron deficiency in crop species.

For Zn and Fe cross-talk, transcriptomic analysis indicates that many Zn uptake- and homeostasis-related genes are up-regulated in Fe-deficient soybean root and leaf (Moran et al., 2014), including those encoding six members of the ZIP gene family, IRT1, the NAS2, and NRAMP3. Similarly, the Fe deficiency responsive *AtIRT1* gene (**Figure 3**) identified could be a key player in the coordination between Zn- and Fe-deficient signaling in *Arabidopsis* (Connolly et al., 2002; Vert et al., 2002; Briat et al., 2015). Furthermore, the vacuolar membrane protein encoding genes *MTP3*, *HMA3*, and *ZIF* essential for Zn tolerance are up-regulated in response to Fe deficiency or Zn excess (Becher et al., 2003; Arrivault et al., 2006; van de Mortel et al., 2006; Haydon and Cobbett, 2007; Haydon et al., 2012). A recent study has confirmed that the MATE transporter gene *FRD3* is involved in cross-talk between Zn and Fe homeostasis for the tolerance to Zn excess in *Arabidopsis* (Pineau et al., 2012), highlighting the complexity of cross-talk between these signaling pathways to regulate Fe deficiency and Zn excess.

Several recent reports have started to discuss the complex tripartite cross-talks among Pi, Zn, and Fe (Briat et al., 2015; Rai et al., 2015). Pi nutrition is affected by the interaction between Zn and Fe in plants. The MYB TF PHR1 apparently acts as a common regulator of Pi, Zn, and Fe homeostasis (**Figure 3**) and functions as a general integrator of multiple nutrition signals (Briat et al., 2015). Firstly, PHR1 was defined as a key regulator for the expression of Pi transporters PHT1 and PHO1;H1 through PHR1–miR399–PHO2 pathway. Secondly, PHR1 seems to be a regulator of the ZIP transporters ZIP2 and ZIP4 for Zn mobilization. In addition, the transcriptional activation of some genes involved in maintaining Fe homeostasis is also shown to be PHR1-dependent manner, including the *FER1* gene encoding the Fe storage protein ferritin, and the *PHO1;1* gene encoding Pi transporter. Saenchai et al. (2016) have reported the OsPHO1;1 is involved in the coordination between Fe transport and Pi–Zn deficiency signaling in rice. Nevertheless, fundamental aspects regulating the cross-talk between Pi, Zn, and Fe deficiency signaling and the regulation of nutritional homeostasis in plants remain to be discovered.

### Pi and Zn Interactions in Mycorrhizal Plants

In the last several decades, the cross-talk between Pi and Zn nutrient homeostasis has been well recognized at the physiological level in many mycorrhizal plants (Reed, 1946; Cakmak and Marschner, 1986; Huang et al., 2000; Watts-Williams et al., 2014; Watts-Williams et al., 2017; Nafady and Elgharably, 2018). High Pi treatment substantially decreased Zn concentration in wheat shoots and grain when these plants were grown in native soils (Ova et al., 2015), and these data also revealed that the negative effect of increasing Pi application on root Zn accumulation and shoot Zn distribution in wheat is dependent on mycorrhization. Furthermore, Zhang et al. (2016) proposed that Pi treatment decreased the Zn concentration in wheat, and they also found that Zn concentration in roots and shoots of maize decreased with increasing Pi supply, and root Zn accumulation exhibits the Pi-induced Zn deficiency during mycorrhization (Zhang et al., 2017), because Pi treatment inhibits colonization resulting in impaired mycorrhizal uptake pathway and then affects the Zn uptake and tissue Zn status of host plants (Loneragan and Webb, 1993; Marschner, 2012; Watts-Williams et al., 2013). The negative relationship between Pi application and the grain Zn status was also confirmed in field studies (Ryan et al., 2008; Zhang et al., 2012). Conversely, under AM conditions, Pi content in shoots of *Medicago truncatula* was greatly reduced when excess Zn was applied in soil (Watts-Williams et al., 2017). Interestingly, an experiment with lettuce plants grown under excessive Zn levels showed that Zn content in mycorrhizal lettuce was greatly reduced when the nutrient solution contained low Pi concentration (Konieczny and Kowalska, 2017). This is indicative of the “protective effect” of arbuscular mycorrhiza, where host plants acquire much less Zn from the Zn excess soils (Chen et al., 2003; Watts-Williams et al., 2013; Christie et al., 2004). Altogether, the interaction between Pi–Zn nutrients during AM symbiosis can be concluded as follows: Crops grown with sufficient Pi decrease Zn in the roots and/or shoots of crops, and inversely, excess Zn reduces Pi in the shoots. However, the underlying molecular mechanisms of the Pi–Zn interaction in mycorrhizal symbiosis are still unclear, and only a few reports discuss the molecular basis of these interactions (Cakmak and Marschner, 1986; Zhu et al., 2001; Watts-Williams et al., 2013; Watts-Williams et al., 2017). Future studies are required to elucidate the molecular basis of the interactions between Pi and Zn nutrient homeostasis during AM symbiosis.

### Pi and Fe Interactions During AM Symbiosis

The antagonistic physiological and molecular interactions between Pi and Fe nutrition have been established in model systems such as *Arabidopsis* and rice (Hirsch et al., 2006; Ward et al., 2008; Zheng et al., 2009; Jain et al., 2013; Rai et al., 2015), but very little information is available on their interactions in mycorrhizal plants.

A couple of studies performed in some edible crop species uncovered the existence of a negative relationship between Pi and Fe uptake in mycorrhizal plants (Azcón et al., 2003; Ferrol et al., 2016; Hoseinzade et al., 2016; Nafady and Elgharably, 2018). Under low Pi supply, the acquisition of Fe increases in mycorrhizal plants (Watts-Williams and Cavagnaro, 2014; Ferrol et al., 2016), and conversely, host plants decrease the Fe accumulation under high Pi conditions during AM symbiosis. Interestingly, Fe content of the straw was greatly increased with low Pi supply during AM symbiosis (Hoseinzade et al., 2016), indicating that mycorrhized rice has reduced Fe nutrient transported to shoots at high Pi status. Very recently, Nafady and Elgharably (2018) reported a similar negative effect of Fe content when maize was treatment with Pi fertilizers. These studies have demonstrated the negative effects of high Pi application in soil on Fe accumulation in mycorrhizal plants (Azcón et al., 2003). Further, the studies have showed the effect of high Pi application on the uptake and transport of Fe nutrition in both rice and maize during AM symbiosis, which could result in the appearance of iron deficiency symptoms under low Fe conditions. However, the effect of Fe treatments on Pi nutrition has not been investigated so far during mycorrhization. The molecular bases of the cross-talk between Pi and Fe in mycorrhizal plants need to be further explored.

### Zn and Fe Interactions in Mycorrhizal Plants

Zinc interacts with some micronutrients such as Fe and copper (Cu) in plants (Poshtmasari et al., 2008; Jain et al., 2013). The cross-talk between the effects of Zn rates on Fe accumulation and translocation has been partially studied in several mycorrhizal plants. Zinc treatment resulted in Fe accumulation in soybean roots under arbuscular mycorrhizal conditions but inhibited Fe translocation from roots to shoots (Ibiang et al., 2017), indicating the cross-talk in Zn and Fe status within the whole soybean during AM symbiosis. However, excess Zn increased root to fruit Fe translocation during AM symbiosis in tomato plants (Ibiang et al., 2018), whereas excess Zn could also lead to a decrease in Fe concentration in mycorrhizal roots. These studies performed under AM conditions have revealed that the physiological antagonistic interaction between Zn and Fe nutrients occurred in roots or shoots depending on the host-plant species. Zn status may therefore affect Fe uptake and transport mechanisms in mycorrhizal plants. These studies have indicated the effect of Zn treatment on the accumulation and homeostasis of Fe nutrition in mycorrhizal plants. However, the effect of Fe availability on Zn nutrition in mycorrhizal plants has not been studied yet, and little information is available on this issue. From the nutritional aspect, there exists a competition between Zn and Fe elements; host plants require coordinate Zn–Fe homeostasis to avoid ion imbalances. Under excess Zn, mycorrhizal plants will decrease the overaccumulation of Fe in shoots prone to Fe starvation. Few studies have identified the potential molecular components involved, and no key genes have been characterized so far acting in the phenomenon. Therefore, the molecular bases of the Zn–Fe interactions in mycorrhizal plants remain largely unknown, and the evidence for the molecular basis of the Zn–Fe co-regulation that mediates the adaptation of a mycorrhizal plant to Zn and Fe availability should be provided in future studies. In particular, the potential genes are involved in the cross-talk between the Zn and Fe homeostasis during AM symbiosis. For instance, the expression of the zinc- and iron-regulated transporter-like proteins (ZRT, IRT-like proteins, referred as to ZIP family) encoding genes in roots and shoots is induced at the transcriptional level by Zn and/or Fe availability (Pedas et al., 2009; Li et al., 2013; Fu et al., 2017), indicating that these ZIP genes may control the uptake and homeostasis of Zn and Fe in mycorrhizal plant species (Grotz et al., 1998; Hall and Williams, 2003).

### Cross-Talk Between Pi–Zn–Fe Nutrient Homeostasis in Mycorrhizal Plants

The above studies provide new insights on genes involved in the potential regulation of nutrient homeostasis in conditions when an individual element is limiting. However, recent research indicated that plant survival is affected by a complex cross-talk between Pi, Zn, and Fe homeostasis (Briat et al., 2015). Interestingly, Saenchai et al. (2016) reported that *OsPHO1;1* was transcriptionally up-regulated in response to Pi–Zn–Fe combined stresses and involved in Fe transport and integrative Pi–Zn deficiency signaling in rice, providing a genetic basis for tripartite Pi–Zn–Fe signaling cross-talks in plants. However, how the members of the plant PHO1-type Pi transporter family function as key linkers in the cross-talks between Pi–Zn–Fe signaling during AM symbiosis has not been elucidated. Although the cross-talks between these nutrients have been touched upon in some model plant studies (Misson et al., 2005; Zheng et al., 2009; Saenchai et al., 2016), the molecular mechanisms of the tripartite interactions during AM symbiosis are still lacking.

## Conclusion

Over the last seven decades, large numbers of studies have focused on how to interpret the potential mechanisms for phosphorus uptake and signaling at molecular and cellular levels in *Arabidopsis* or rice. The combination of molecular and cellular biology, multiple “omics” approaches, and reverse genetics has resulted in the characterization of many important genes that control Pi accumulation and homeostasis in *Arabidopsis* and rice in response to Pi limitation. However, Pi is well known to interact with some micronutrients such as Zn and Fe in plants (Bouain et al., 2014a; Briat et al., 2015). Future research will need to undertake an integrative study to uncover the defined mechanisms by which plants coordinate the Pi, Zn, and Fe deficiency signaling in order to enhance their fitness during multiple Pi, Zn, and Fe deficiency stresses. In such a context, the principal aim of this review is to broaden the current understanding of the cross-talk between the Pi and Zn, Pi and Fe, Zn and Fe, and Pi–Zn–Fe homeostasis in nonmycorrhizal and mycorrhizal plants. In addition, the identification of important genes regulating the interactions between Pi, Zn, and/or Fe transport and signaling in plants, particularly in crop species, will help breeders develop new strategies for nutrient management, and taking into account the interactions between plants and their AM fungal symbionts. In conclusion, the development of the integrative study of cross-talk between Pi, Zn, and Fe signaling pathway will be of great interest and essential for sustainable agricultural development all around the world.

## Author Contributions

MT and XX conceived and designed this study. XX, WH, and XF wrote the manuscript. HC proposed related theories and assisted with the interpretation of some references. All authors have read, edited, and approved the current version of the manuscript.

## Funding

This work was supported by grants from the National Natural Science Foundation of China (grant no. 31800092), the Natural Science Foundation of Guangdong Province in China (grant no. 2018A030313141), the Key Projects of Guangzhou Science and Technology Plan (grant no. 201904020022), and the High-level Talent Start Funding Project of South China Agricultural University (grant no. 218066).

## Conflict of Interest

The authors declare that the research was conducted in the absence of any commercial or financial relationships that could be construed as a potential conflict of interest.
